# Treatment of recurrent aphtous stomatitis: A systematic review

**DOI:** 10.4317/medoral.25604

**Published:** 2022-09-29

**Authors:** F Javier Parra-Moreno, Sonia Egido-Moreno, Mayra Schemel-Suárez, Beatriz González-Navarro, Albert Estrugo-Devesa, José López-López

**Affiliations:** 1 Master Student. Master of Oral Medicine, Surgery and Implantology, Medicine and Health Sciences Faculty, UB, Barcelona, Spain; 2 Stomatology Department, Barcelona University. Master of Oral Medicine, Surgery and Implantology, Medicine and Health Sciences Faculty, UB-Dental Hospital, Barcelona, Spain; 3 Stomatology Department, Barcelona University. Master of Oncologic and Immunocompromised patients, Medicine and Health Sciences Faculty, UB-Dental Hospital, Barcelona, Spain; 4 Stomatology Department, Medicine and Health Sciences Faculty, Dentristry. Research group in Oral Health and Masticatory System, Biomedical Investigation Centre of Bellvitge (IDIBELL), L´Hospitalet de Llobregat, Barcelona, Spain. Dental Hospital of the University of Barcelona, University of Barcelona, Barcelona, Spain; 5 Faculty Director and Head of Service of the Medical-Surgical Area of Dentistry Hospital, University of Barcelona, Barcelona, Spain

## Abstract

**Background:**

Recurrent Aphtous Stomatitis (RAS) is the most common process affecting the oral mucosa. It is painful, multifactorial and generally recurrent. The aim of this systematic review is to know the last treatment approaches and their effectivity.

**Material and Methods:**

we compared the outcome of different kind of treatments in terms of the improvement of the lesions, reduction of the size of those lesions and the time needed for their healing. Inclusion criteria were: clinical trials, articles written in English or Spanish and published less than 5 years ago.

**Results:**

we used the following keywords: “treatment”, “aphtous stomatitis”, “canker sores”; combined with Boolean operators AND y OR. We selected 28 articles for reading the whole text, and after applying the eligibility criteria, we selected 17 articles for our revision. Among all the treatments, we emphasize the barrier method based in compound of cellulose rubber and a calcium/sodium copolymer PVM/MA, with which the difference in the 3rd and 7th day was of -6,29 ± 0,14 points in the pain score. The treatment with insulin and chitosan gel, brought a pain suppression on the third day, with no reactivation of the pain during the whole study. The application of a film composed of polyurethane and sesame oil with chitosan, brought a reduction in the size of the lesions of 4,54 ± 2,84mm on the 6th day compared with the situation before the beginning of the treatment. The different kinds of laser, which produced a reduction in the pain score just at the beginning of the treatment up to 8,1 ± 1,6 points, and a reduction of the size of the lesions of 4,42 ± 1,02mm on the 7th day.

**Conclusions:**

Besides the classic treatments for RAS, we have to take into account other treatment modalities, above all the different kinds of laser.

** Key words:**Recurrent aphtous stomatitis, canker, treatment, food supplements, topical treatment, systemic treatment.

## Introduction

Recurrent Afthous Stomatitis (RAS) is the most common inflammatory process of the oral mucosa. Furthermore, it is painful, multifactorial and generally recurrent (1,2). It is characterized by its periodicity and for being self-limited. Its prevalence ranges between 5 and 25% of the population (1,3). RAS manifests by the appearance of one or several painful ulcerations (cankers), covered by a white or greyish pseudomembrane and surrounded by a well defined erythematosus halo. Lesions are usually located on the non-keratinized oral mucosa and they can present recurrences after variable remission periods (4).

Based on the lesions size, number and duration, RAS can be classified into three types: major, minor and herpetiform (5). Minor canker sores suppose the 80-90% of all RAS lesions. These lesions are typically smaller than 1cm of diameter and they heal in a period of 7-14 days without leaving any scar. The major lesions are the 10-15% of all the lesion, they are bigger than 1cm of diameter and heal between 20-30 days, leaving scars. The herpetiform lesions are present in a 5-10% of cases. The lesions are multiple and grouped. They have a size between 1-3mm, and usually coalesce in bigger lesions and could need up to 15 days for their healing (4-8).

These ulcers have an unclear etiology, but some factors as heredity, immune disorders, hematological deficiencies (like iron, folic acid, vitamin B6 and B12), stress, local traumas, infections and systemic infections (Behçet Syndrom) are considered predisposing factors (9-11).

Histology of these lesions show an epithelial ulceration, with an exudate over its surface, a necrotic tissue, an inflammatory cells infiltrate, oedema of the lamina propria with diverse degrees of neutrophils and mononuclear cells infiltration, as well as a hyaline degeneration. There is a number of inflammatory cells surrounding the blood vessels. We can also see an expansion and a capillary congestion, an enlargement of vascular endothelial cells and a narrowing of lumen of these vessels. This inflammatory process plays an important role in the onset of RAS (1,12,13).

Pain can be derived from excessive inflammation and chemical irritation of the afferent never endings in the epithelial and subepithelial layers junctions. This pain can hinder common actions as chewing, speaking, swallowing, apart from affecting the patient´s quality of life (1,12,13).

Taking into account that the specific etiology of RAS is still unknown, its management is still focused on relieving the symptoms. The objective of the treatment, therefore, is to reduce de inflammation, reduce the pain, extend the periods between disease outbreaks and promote the healing. There have been many treatment approaches, however topical corticosteroids are still the gold standard. They have shown a beneficial effect in reducing the pain and duration of the ulcers. Nevertheless, their continue and inappropriate use can lead to adverse effects, mainly related to a possible systemic absorption of the drug (1,14).

The aim of this systematic review is to know the last approaches in terms of treatment for RAS and their effectiveness against signs and symptoms of the disease, as well as the reduction in the time needed for the healing of the lesions.

## Material and Methods

The authors followed the guidelines for systematic revisions according to PRISMA statement (15), and also followed the Grading of Recommendations, Assessment, Development and Evaluation (GRADE) (16), in order to analyze the degree of evidence.

-Search strategy

We performed a search in PubMed data base limited to the last 5 years, using these key words “treatment”, “aphtous stomatitis”, “canker sores”; combined with Boolean operators AND and OR.

-Eligibility criteria

For the papers selection we used the question problem-intervention-comparation-results (PICO): we wanted to compare the results related to the improvement of RAS lesions, taking into account the pain scales, the reduction of the size of the lesions and the time needed for their healing. The papers compared different treatment strategies with a placebo or two different treatments.

The data we registered were age and sex of every patient, the country of origin of every study, the therapeutic options of the cases, the pain score in every visit, as well as the size of the lesions and the days needed for their healing.

-Inclusion and exclusion criteria

The inclusion criteria were: clinical trials, articles written in Spanish or English and no more than 5 years since their publication. The exclusion criteria were: articles which were not available in English or Spanish, which sample consisted in patients with previous pathologies an articles with no information regarding to age or sex of patients.

The variables examined are pain reduction, decrease in the size of the lesions and days needed for their healing.

## Results

-Selection of studies

**Figure 1:**
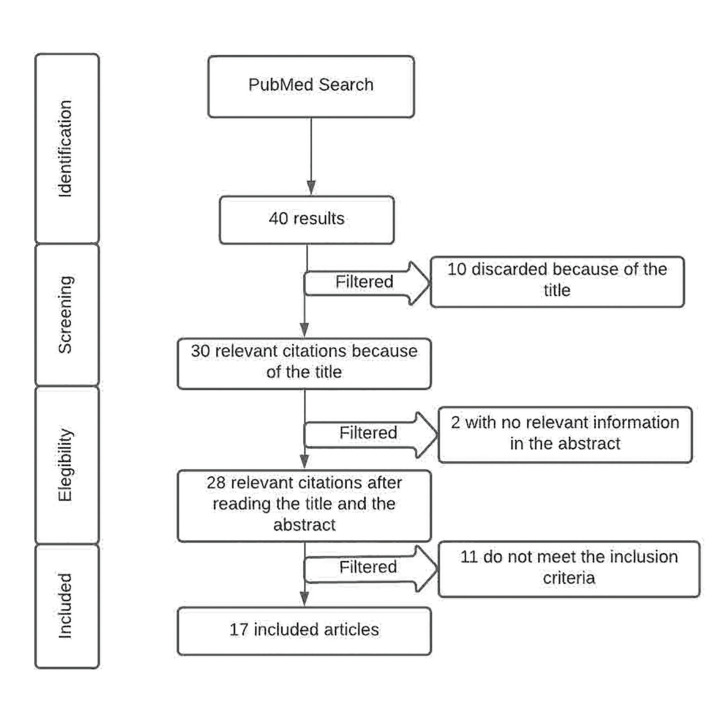
Flow chart of the selection of articles: RAS treatments.

We show a flow chart which describes the identification, inclusion and processes of exclusion of those studies which were selected (Fig. 1). The studies selection was performed in three stages. On the 1st stage, after the first bibliographic search we obtained 40 articles, of which after reading the title and eliminate the duplicate articles, we discarded 10 articles. On the 2nd stage, of the 30 papers selected we discarded 2 articles because they did not have relevant information in their abstracts. On the 3rd stage, we selected 28 articles for the revision of the whole text, we applied the eligibility criteria, inclusion and exclusion. Finally, we selected 17 articles that met the inclusion criteria for our revision (1-3,5,11,12,14,17-26).

This systematic review fulfill 21 of 27 items of PRISMA statement (15) and the degree of evidence is high (16).

-Studies features (Table 1)

All the 17 articles included in this review were written in English and Spanish and were published between 2016 and 2021 (1-3,5,11,12,14,17-26). Altogether there were 985 patients examined, divided in 505 cases and 480 controls. The proportion of men was 45,28% and 54,72% of women. The number of cases for study was from 19 (20) to 140 (12).

Regarding the geographic distribution, 10 of the papers were published in Asia (2 from Iran, 1 from Israel, 1 from Turkey, 4 from China and 1 from India) (1,3,11,12,14,21,23-26). 4 published in Europe (2 from Italy, 1 from Denmark and 1 from Liechtenstein) (5,17,19,20). 3 published in Africa (all them from Egypt) (2,18,22).

We divided the studies in the kind of treatment used and the patients were distributed in “cases” if they received the experimental treatment, and “controls” if they received a placebo or another treatment in order to compare their efficacy. According to that, we divided the treatments obtained in three categories: “food supplements” (3,17,18) with 65 cases and 65 controls, “topical treatments” (1,2,5,11,12,14,19-25) which included 408 cases and 387 controls. Finally, the group of “systemic treatments” (26), with only one article with 32 cases and 28 controls.

When we grouped all the cases, there were 45,35% of men and 54,65% of women, while in the control group there were 45,21% of men and 54,79% of women.

The average age of all the subjects in this revision is 28,76 ± 6,21 years, being 28,76 ± 6,21 years in the cases group and 28,44 ± 5,84 years in the control group. In Akbari´s *et al* (23) work, patients were divided in age ranges. The most numerous group was the one between 20 and 50 years, with 82,85% of cases and 85,71% of controls. Likewise, Huo *et al* (24), classified their patients in two groups: less of 40 years old and more than 40 years old. In the first group there are 64% of cases and 50% controls and in the group of more than 40 years old, there are 36% of cases and 50% of controls (Table 1).

**Table 1:**
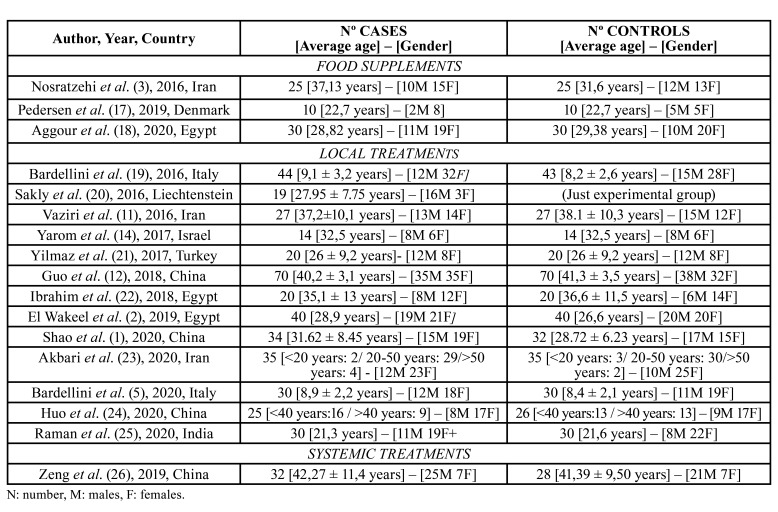
Demographic data of the subjects included in every study and year of publication.

-Summary of results

Among all the pain scales, the majority of studies use VAS (Visual Analogic Scale). In that scale, the patient has to give a score of the pain from 0 (no pain) to 10 (maximum pain) (1-3,5,11,12,17-21,23-25,26). Ibrahim *et al* (22) use the scale OCMI (Oral Clinical Manifestations Index) which combines different factors, giving a score to every one of them. These factors are: the type of lesions (minor, major or herpetiform), number of lesions, duration of every outbreak, frequency of the outbreaks and the score given to the pain taking into account interferences with chewing.

The results of the studies are detailed in Table 2, Table 3 and Table 4.

**Table 2:**
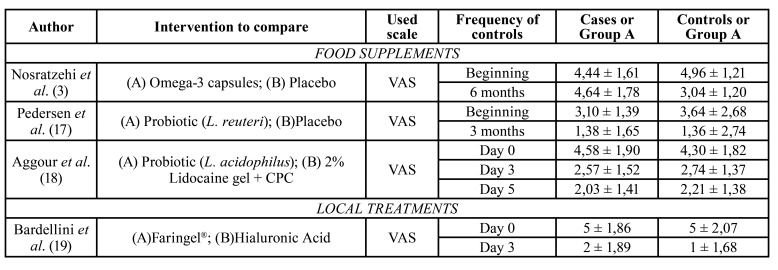
Arithmetic means of pain scales of the subjects included in every study, in every revision visit.

**Table 2 cont.**
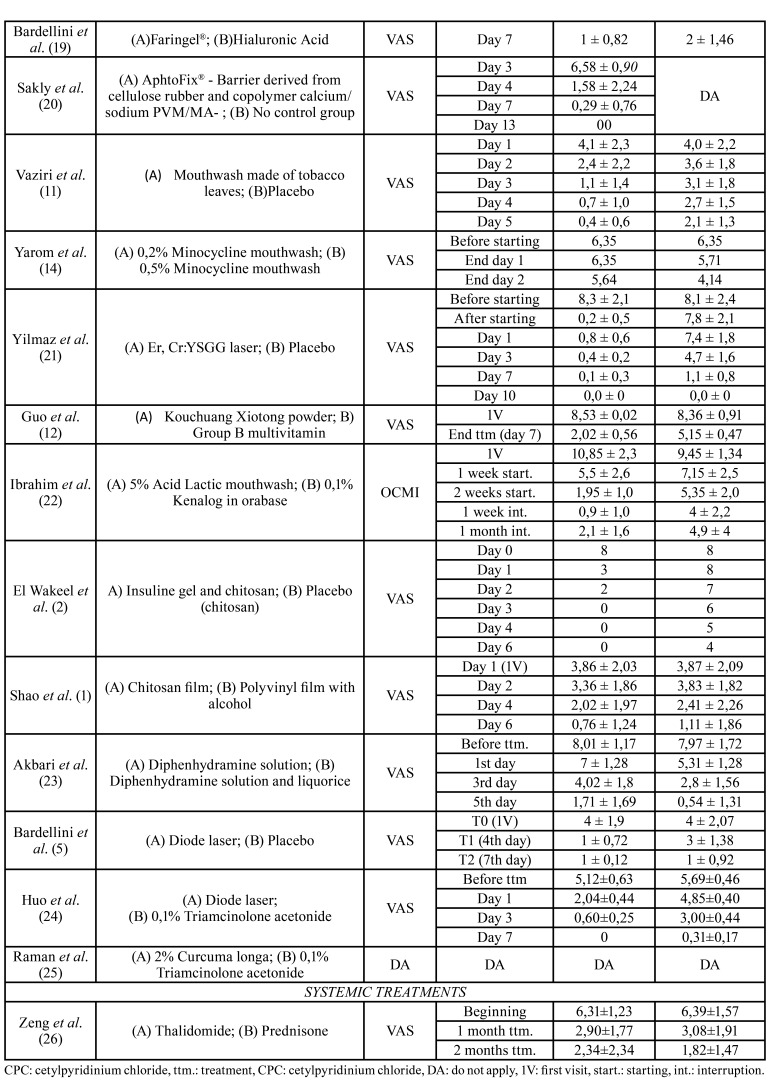
Arithmetic means of pain scales of the subjects included in every study, in every revision visit.

**Table 3:**
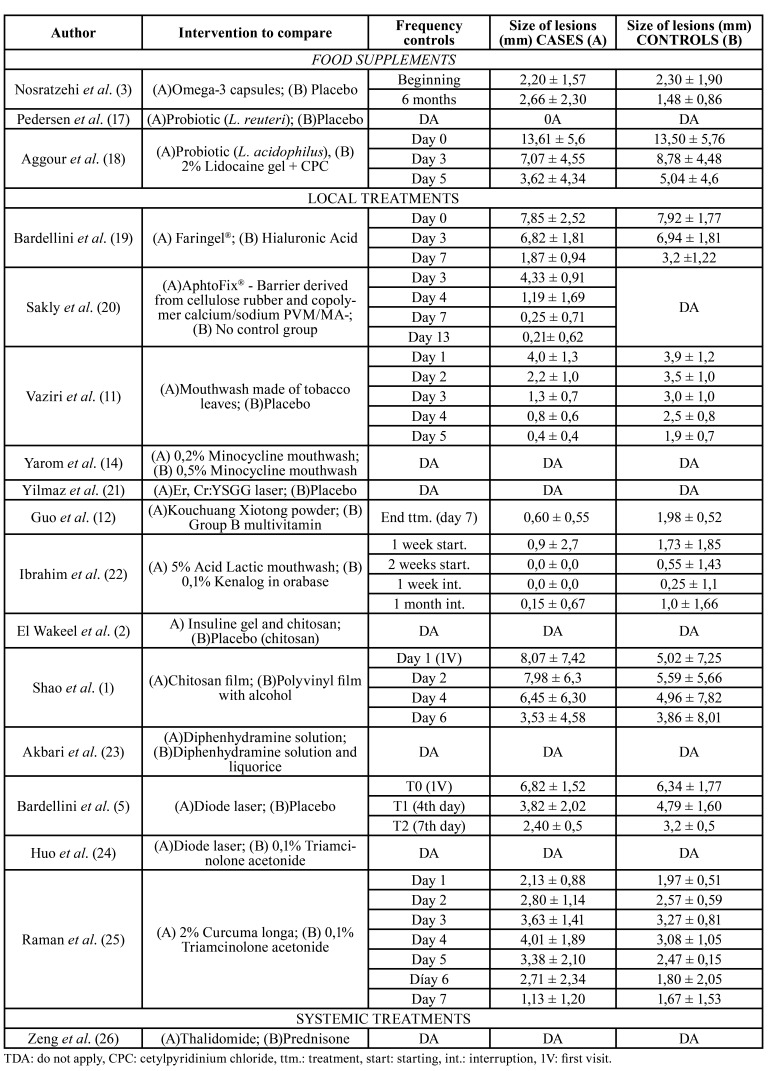
Arithmetic means of included subjects´ size of lesions (major diameter), in every revision visitand arithmetic means of days needed for lesions healing of subjects included in every study.

**Table 4:**
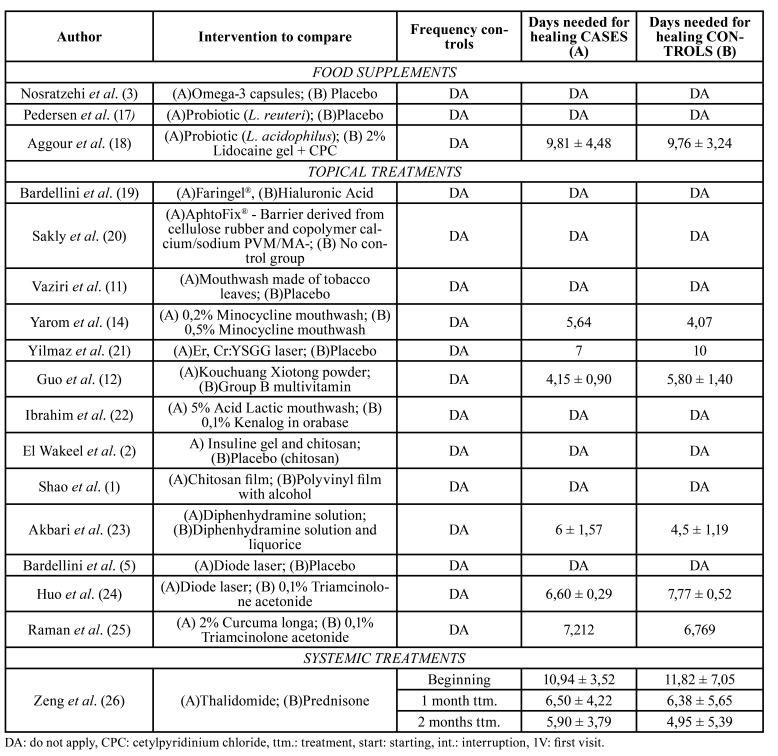
Arithmetic means of days needed for lesions healing of subjects included in every study.

-Food supplements

Nosratzehi *et al*. (3) compared the administration of 1.000mg of Omega-3, 3 times a day for 6 months. The symptomatology reduction at 6 months was of 1,72 ± 0,26 points in Omega-3 group, unlike the difference in placebo group which is -1,92 ± 0,01 points. There was a small increase of 0,46 ± 0,73mm in the size of the lesions at 6 months of treatment in the study group; while in the control group there was a reduction of 0,82 ± 1,04mm.

Pedersent *et al*. (19), compared the use of probiotic twice a day during 90 days with a placebo, while Aggour *et al* (18) compared the administration of a probiotic with the compound made of 2% lidocaine gel and cetylpyridinium chloride, both twice a day during 5 days. In both studies there were not significative differences between groups.

Aggour *et al*. (18), saw a reduction of the size of the lesions in the study group of 9,99 ± 1,26mm, on the 5th day; while the reduction of the control group was of 8,46 ± 1,16mm. There were not statistical differences in the time needed for the healing of the lesions.

-Local treatment

Bardellini *et al*. (19) compared the product Faringel®, which is composed by sodium bicarbonate and alginate, aloe vera, propolis, chamomile, calendula and honey; with hyaluronic acid. Both applied 3 times a day for 7 days. The group which used Faringel® presented a pain reduction of 3 ± 0,03 points, while the hyaluronic acid promoted a pain reduction of 4 ± 0,39, both on the 3rd day of treatment. Nevertheless, on the 7th day, in the Faringel® group the reduction was 1 ± 1,07 points and hyaluronic acid gave a rise in the pain score of 1 ± 1,22 compared with the results on the 3rd day. The reduction of the size of the lesions on the 7th day was 5,98 ± 1,58mm in the Faringel® goup and 4,72 ± 0,55mm in the hyaluronic acid group.

Sakly *et al*. (20), examined the effect of Aphtofix® applied on the ulcers. That gel is composed by cellulose gum and copolymer of calcium/sodium PVM/MA. This product had to be applied 4 times a day, for 14 days. The difference between the 3rd and 7th days was -6,29 ± 0,14 points of pain. There was a reduction of the size of the lesions of 4,08 ± 0,2mm, on the 7th day.

Vaziri *et al*. (11) compared a mouthwash composed of tobacco leaves with a placebo. Both administered 3 times a day during 5 days. On the 5th day, there was a reduction of symptomatology of 3,7 ± 1,7 points in the study group, while in the placebo group, this reduction was 1,9 ± 0,9 points. The reduction of the size of the lesions on the 5th day was of 3,60 ± 0,90mm in the study group and 2,00 ± 0,50mm in the placebo group.

Yarom *et al*. (14), compared two different concentrations of a mouthwash composed of minocycline: 0,2% and 0,5%. They were used 4 times a day, since the outbreak of the lesions until they disappeared, with a maximum of 10 days. At the end of the 2nd day there was a reduction of 0,71 points of pain in the group with a concentration of 0,2% and 2,21 points in the group with concentration of 0,5%. The group with the concentration of 0,5% needed 1,57 less days for the healing of lesions.

Yilmaz *et al* (21), used the Er,Cr:YSGG laser, 20 seconds in the first visit. The VAS scale gave an average of -8,1 ± 1,6p points just after starting the treatment, while in the placebo group, that difference was of -0,3 ± 0,3 points. On the 7th day the difference was -8,2 ± 1,3 points in the study group and -7 ± 1,6 points in the placebo group. It is worth stressing that on the first day there is a rise of 0,6 ± 0,1 points in the pain scale in the laser group.

In Ibrahim *et al*.´s study (22), a group of patients used a mouthwash of 5% lactic acid 3 times a day for 1-2 weeks. There was a difference of -9,95 ± 1,3 points on the OMIC scale after a week of having interrupted the treatment in the study group, compared with the first visit. The control group used Kenalog® (0,1% triamcinolone acetonide with orabase) twice a day during 1 or 2 weeks. This group presented a reduction of the symptomatology of 3,1 ± 1,2 points compared to the study group, after a week of interruption of the treatment. The reduction of the size of the lesions after one week of interruption of the treatment was 0,9 ± 2,7mm in the study group, while the reduction was 1,48 ± 0,75mm in the Kenalog® group (they did not take into account the size of the lesions on the 1st visit in order to compare it).

El Wakeel *et al*. (2) used a gel with insulin and chitosan once a day over the lesions on the study group, while in the control group they used just chitosan. In the study group, VAS scale presents a reduction of -8 points (total reduction) on the 3rd day, maintaining it the rest of the study, while in the other group there is a reduction of 2 points on the same day.

Shao *et al*. (1), compared the use of a polyurethane film with a sesame oil and a chitosan layer, versus a polyvinyl film with alcohol. There were not significative differences in terms of relieving the pain in both groups. However, the reduction in the size of the lesions was 4,54 ± 2,84mm in the group with chitosan on the 6th day, and 1,16 ± 0,76mm in the group of the film impregnated with alcohol.

Akbari *et al* (23), contrasted the use of a diphenhydramine solution and a diphenhydramine solution mixed with licorice. There was a reduction of the pain of 3.99 ± 0,63 on the 3r day in the 1st group, and a reduction of 5,17 ± 0,16 in the group with licorice. In this second group, the lesions healed 1,5 ± 0,38 days earlier than in the 1st group.

Bardellini *et al*. (5), compared a diode laser with a placebo. The laser was applied during the 1st day and every day the 3 next days. There was a reduction of the pain sensation of 3 ± 1,18 points on the 4th day with the laser group and that reduction was 1 ± 0,69 points in the placebo group. The reduction in the size of the lesions in the laser group was 4,42 ± 1,02mm on the 7th day, while in the placebo group it was 3,14 ± 1,27mm.

Huo *et al*. (24), contrasted the same laser than the previous study with the use of an ointment of 0,1% triamcinolone acetonide. They applied them every day for 3 days. In the laser group there was a reduction of pain of 4,52 ± 0,38 points on the 3rd day, compared with previous situation. The symptomatology disappeared on the 7th day for this group. In the group using a triamcinolone acetonide, the pain reduction was 2,69 ± 0,02 points on the 3rd day. In the laser group the lesion healed 1,17 ± 0,23 days before compared with triamcinolone acetonide group.

Raman *et al*. (25), compared the use of an ointment with 2% Curcuma Longa and 0,1% acetonide triamcinolone, both taken 3 times a day till the symptomatology eased completely. The reduction of the size of the lesions on the 7th day in the first group was 1 ± 0,32mm, while in triamcinolone acetonide group, the reduction was 0,3 ± 1,02mm. There were not statistically significative differences in the time of healing of the lesion.

-Systemic treatments

We just found one study which compared the treatment with Thalidomide and the treatment with Prednisone, both taken orally. The posology for thalidomide was: 100mg daily for 10 days, followed for 50mg daily for 10 days, finishing with 25mg daily for the next 10 days. Prednisone was applied 0,4mg/kg daily for 15 days, followed by 0,2mg/kg daily for the next 15 days There was a reduction of the symptomatology in the first group of 3.97 ± 1,11 points and 4,57 ± 0,1 points in the second group. Lesions healed 0,95 ± 1,6 days before in the group with prednisone (26).

## Discussion

RAS is a common oral condition which is characterized by a recurrent appearance of usually little ulcers. These lesions are ovoid or rounded, not infectious, not vesicular and they are immunologically mediated, with limited margins which are surrounded by a erythematous halo (21).

The aim of the treatments are the reduction of that inflammatory response, the pain relief, the enlargement the intervals between outbreaks and the promotion of the healing (1).

Nowadays, therapeutic approaches depend on the severity of the lesions, their frequency and the duration of the outbreaks. They also depend on the patients´ medical history and their capacity to tolerate that medication. Taking all these points into account, the treatment has been divided in 5 phases or steps. The first ones are the general measures which include improvement of the oral hygiene, food supplements as omega 3 o different probiotics. The second step are the topical treatments, as barrier measures, topical anesthetics, amlexanox or topical corticosteroids. Next ones are first line systemic treatments, as systemic corticosteroids and second line systemic treatments, such as thalidomide, dapsone, montelukast, among others. Finally, some biological treatments have been proposed, some examples are pentoxifylline, etanercept and adalimumab (27).

In our revision we divided the different treatments found in the literature in food supplements, topical treatments and systemic treatments.

Among food supplements, probiotic treatments exert a wide range of actions on a number of immunological cells, taking them to an anti-inflammatory action. They modulate the mucosal immunological mechanism, through the reduction of some proinflammatory cytokines by means of actions on NF𝛋B (nuclear factor Kappa B) pathway, increasing the production of some other proinflammatory cytokines as IL-10 and defensive peptides as b-defensine, improving the protection of IgA, influencing the maturation of dendritic cells and promoting the activity of the regulatory T-lymphocytes. Furthermore, the probiotics actions can be boosted by the use of prebiotics as inulin. This relationship is known as symbiotic (17,18). In this revision, food supplements do not present any benefit over placebo or anesthetic gels in terms of pain reduction or days needed for lesions healing. Nevertheless, some papers say there is a reduction of more than 1mm in the size of the lesions on the 3rd and 5th days (3,17,18).

Minocicline is an antibiotic of tetracyclines group. The rationale for its use is the presumption that RAS has a local infection origin. Nevertheless, it has shown good results in controlling cutaneous non-infectious diseases as bullous and scar-like pemphigoid, and lineal IgA dermatosis. It has been hypothesized that minocycline has a cytokines regulatory effect which can be related to development of RAS. In our work, they compared two concentrations (0,2% and 0,5%). The higher concentration showed greater advantages in terms of pain reduction and reduction of the days needed for the healing of the lesions (14).

The different kinds of laser (for instance Er,Cr: YSGG, Nd: YAG, CO2, diode, etc) also bring a local action on the area where they are applied. It has been demonstrated that they accelerate the healing of the lesions, since they promote re-epithelialization of the ulcers, proliferation of fibroblasts, synthesis of collagen, increase the vascularization of the area and reduce alterations of nerve impulse conduction. In our revision, laser provides a considerable reduction of symptomatology, of the size of the lesions since the starting of the treatment, and the time needed for healing (5,21,24).

Ibrahim *et al* (22), compared the topical action of lactic acid and triamcinolone acetonide. The first one, increases spontaneous secretion of endothelial vascular growing factor, which is a multifunctional angiogenic cytokine involved in angiogenesis and wounds healing. Furthermore, promotes collagen and elastic fivers production. It also has an immunomodulatory action, reversing the decreasing ratio CD4/CD8 ratio in the aphthous ulcers. It provides an antibacterial action against *Streptococcus* sanguis, which is considered a pathogenic agent associated to RAS. On the other hand, it also shows a local analgesic action. In our work, it shows a considerable pain reduction in all the control visits, comparing it to 0,1% triamcinolone acetonide. Similarly, lesions had completely healed after two weeks of treatment, while they had not completely healed after two weeks of treatment, nor after one week after interrupting the treatment with triamcinolone acetonide.

Triamcinolone acetonide is a synthetic corticosteroid which possesses two pathways of action: anti-inflammatory activity which reduces progression of the ulcers and patient´s discomfort, and on the other hand it blocks interaction of epithelial T-lymphocytes. Given the fact that concentration of sensitized lymphocytes is higher in early stages of oral ulcers, this drug has a bigger effect during this period (22). In our revision, there was a reduction of 5 points of pain on the first day, compared with placebo and there was a complete disappearance of pain after 3 days of use of the medication.

Another gel also used in the literature is the insulin one. Insulin has a capacity to improve the healing through the increase of the ratios of re-epithelialization, angiogenesis and extracellular matrix secretion by keratinocytes, endothelial cells and fibroblasts. Moreover, it has the ability of restoring collagen synthesis and the production of granulation tissue in the initial stages of healing. Nevertheless, when it is applied over a wound, the environment forces it to detach quickly, that is why it has to be applied with high frequency to maintain the therapeutic concentrations. Certain liberation systems as liposomes counteract this fact and have achieved a controlled liberation on the wound site. Liposomes are biocompatible, biodegradable and enable a sustained liberation of the compound (2).

Licorice (Glycyrrhiza glabra) is one of the oldest medicinal plants, already described by Avicenna. Its action is based on the inhibition of cyclo-oxygenase 5 and lipoxygenase enzymatic pathways. It prevents oxidative compounds synthesis and cellular migration, therefore, it inhibits arachidonic acid metabolism and vascular permeability, which decreases inflammatory response. It has also been reported antibacterial and antiviral responses. There has also nor been found complications because of their topical action, however, the big doses taken orally, can increase arterial pressure given its mineralocorticoid effect (23). In our review, there is a comparation of the effect of diphenhydramine with and without licorice. There was an improvement of the symptomatology and the time needed for healing of the lesions in the group with licorice in the solution.

Diphenhydramine is a receptors H1 antagonist with local anesthetic effects which can be used alone or in a combination with other medications as a mouthwash to reduce the pain of the ulcers (23).

Among systemic treatments, oral corticosteroids are considered, according to literature, the first line of them. They have been used with great results both in long term patterns with low dose (oral prednisone 5mg/day for 3 months) or in short term treatments with higher dose (oral prednisone 20-40mg for 4-7 days) with a subsequent progressive decrease of the dose. With those drugs we can achieve a relief of pain, an acceleration of the ulcers healing and a reduction in the number of outbreaks (26,27).

Thalidomide has shown an effect in regulating lymphocytic function and in reducing the concentration of tumoral necrosis factor-alfa (TNF-𝛂). Thalidomide can relieve the pain produced by RAS and can reduce the number of lesions. Literature classifies it as a second line systemic treatment, that is to say, useful in those patients who do not respond in a favorable way to intermittent treatments with systemic corticosteroids, who require longer or more frequent treatments with systemic corticosteroids or those patients in which cannot take corticosteroids because of different reasons. In our review, systemic prednisone offers more beneficial effects in terms of reduction of symptoms and necessary time for healing compared with systemic thalidomide (26,27).

**Figure 2:**
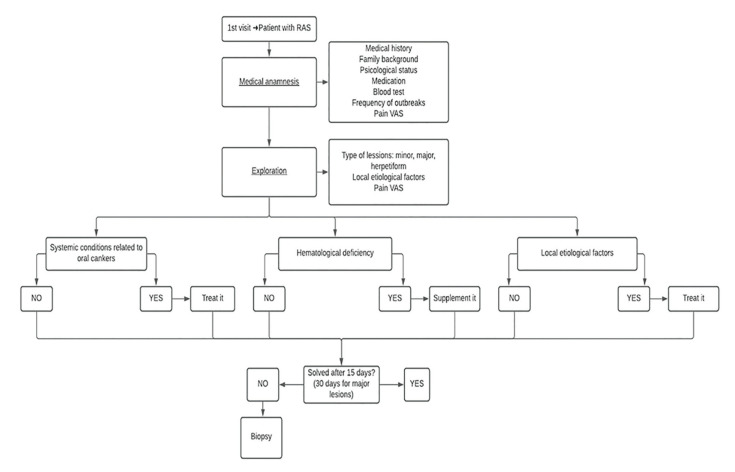
Flowchart summarizing the etiological factors for RAS and what to do in every case.

**Figure 3:**
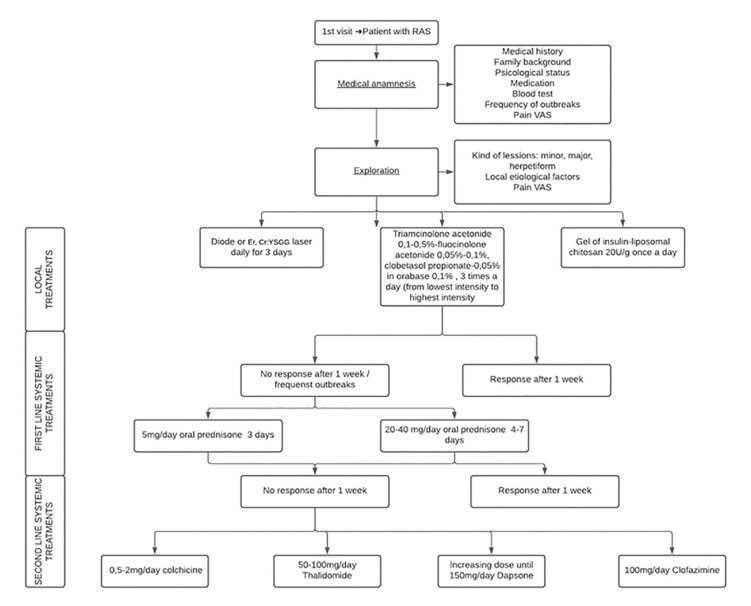
Flowchart summarizing the different modalities of treatment available for these patients.

Based on our search, we have provided a flowchart related to etiological factors for RAS (Fig. 2) and another one summarizing the treatment available for these patients (Fig. 3).

The main limitations of this review is the risk of bias between studies because of different number of patients and different ages, the low data related to systemic treatments and other local treatments recently reported (16).

## Conclusions

RAS is a common condition among general population. It has a multifactorial etiology and we do not have a clear protocol to treat it and, furthermore, it can hinder patients´ usual activities (speaking, chewing and swallowing). These patients have been traditionally successfully treated with topical corticosteroids, nevertheless, in the last years, they have appeared other treatment approaches also effective as barrier methods based on biocompatible polymers, lactic acid mouthwashes, gel made of insulin or diphenhydramine together with liquorice solution. All these new strategies need to be taken into account. Nevertheless, different kinds of laser have shown great efficacy since the beginning of the treatment, with a pain reduction and a decrease of the size of the lesions and the days needed for the healing of the lesions.
